# Cortical and subcortical changes in resting-state neuronal activity and connectivity in early symptomatic ALS and advanced frontotemporal dementia

**DOI:** 10.1016/j.nicl.2022.102965

**Published:** 2022-02-12

**Authors:** Rosanne Govaarts, Emma Beeldman, Matteo Fraschini, Alessandra Griffa, Marjolein M.A. Engels, Michael A. van Es, Jan H. Veldink, Leonard H. van den Berg, Anneke J. van der Kooi, Yolande A.L. Pijnenburg, Marianne de Visser, Cornelis J. Stam, Joost Raaphorst, Arjan Hillebrand

**Affiliations:** a Amsterdam University Medical Centers, University of Amsterdam, Department of Neurology, Amsterdam Neuroscience, Amsterdam, the Netherlands; b University of Cagliari, Department of Electrical and Electronic Engineering, Cagliari, Italy; c Department of Clinical Neurosciences, Division of Neurology, Geneva University Hospitals and Faculty of Medicine, University of Geneva, Geneva, Switzerland; d Institute of Bioengineering, Center of Neuroprosthetics, École Polytechnique Fédérale De Lausanne (EPFL), Geneva, Switzerland; e Amsterdam University Medical Centers, Vrije Universiteit Amsterdam, Department of Clinical Neurophysiology, Magnetoencephalography Centre, Amsterdam Neuroscience, Amsterdam, the Netherlands; f University Medical Centre Utrecht, Department of Neurology, Brain Centre Rudolf Magnus, Utrecht, the Netherlands; g Amsterdam University Medical Centers, Vrije Universiteit, Alzheimer Center, Amsterdam Neuroscience, Amsterdam, the Netherlands

**Keywords:** Amyotrophic lateral sclerosis, Behavioural variant frontotemporal dementia, Magnetoencephalography, Resting-state, Oscillatory brain activity, Functional connectivity

## Abstract

The objective of this study was to examine if patterns of resting-state brain activity and functional connectivity in cortical and subcortical regions in patients with early symptomatic amyotrophic lateral sclerosis (ALS) resemble those of behavioural variant frontotemporal dementia (bvFTD). In a cross-sectional design, eyes-closed resting-state magnetoencephalography (MEG) data of 34 ALS patients, 18 bvFTD patients and 18 age- and gender-matched healthy controls (HCs) were projected to source-space using an atlas-based beamformer. Group differences in peak frequency, band-specific oscillatory activity and functional connectivity (corrected amplitude envelope correlation) in 78 cortical regions and 12 subcortical regions were determined. False discovery rate was used to correct for multiple comparisons. BvFTD patients, as compared to ALS and HCs, showed lower relative beta power in parietal, occipital, temporal and nearly all subcortical regions. Compared to HCs, patients with ALS and patients with bvFTD had a higher delta (0.5–4 Hz) and gamma (30–48 Hz) band resting-state functional connectivity in a high number of overlapping regions in the frontal lobe and in limbic and subcortical regions. Higher delta band connectivity was widespread in the bvFTD patients compared to HCs. ALS showed a more widespread higher gamma band functional connectivity compared to bvFTD. In conclusion, MEG in early symptomatic ALS patients shows resting-state functional connectivity changes in frontal, limbic and subcortical regions that overlap considerably with bvFTD. The findings show the potential of MEG to detect brain changes in early symptomatic phases of ALS and contribute to our understanding of the disease spectrum, with ALS and bvFTD at the two extreme ends.

## Highlights

• Lower resting-state relative beta power in bvFTD compared to ALS and healthy controls.
• Higher delta and gamma band resting-state functional connectivity in ALS and bvFTD patients.
• This functional connectivity overlaps in frontal, limbic and subcortical regions.
• MEG can detect changes in neuronal activity in early symptomatic phases of ALS.
• There is electrophysiological evidence of the disease spectrum of ALS-bvFTD.

## 1 Introduction

Amyotrophic lateral sclerosis (ALS) is a neurodegenerative disorder characterised by progressive muscle weakness leading to death in 3–5 years (Chiò et al., 2009). In approximately 10–40% of the patients, ALS is associated with either the behavioural variant of frontotemporal dementia (bvFTD) or less severe cognitive and/or behavioural changes (Burrell et al., 2016, Lillo et al., 2012, Strong et al., 2017). Although debated by some, ALS and bvFTD are considered to be part of a disease spectrum, with ALS on one end of the spectrum and bvFTD on the other end, which is reflected by similar brain imaging abnormalities and pathological findings beyond the motor cortex (Burrell et al., 2016, Lillo et al., 2012, Strong et al., 2017, Trojsi et al., 2018).

Whereas non-motor symptoms of ALS may be present early in the symptomatic disease course, the reliable detection of these symptoms can be challenging in this relentlessly progressive motor disorder (Turner et al., 2009, Westeneng et al., 2018). The identification of biomarkers reflecting involvement of non-motor areas in early symptomatic phases of ALS is a priority for future clinical trials, in particular to establish the accurate diagnosis as early as possible (Turner et al., 2009). Our understanding of brain dysfunction and related biomarkers within the presumed disease spectrum of ALS-bvFTD will likely increase by an approach that examines patients at both ends of the symptomatic disease spectrum, i.e., early symptomatic ALS patients and advanced bvFTD (Burrell et al., 2016).

Electrophysiological signals reflecting brain (dys)function can be measured by resting-state electroencephalography (EEG) and magnetoencephalography (MEG). In patients with bvFTD and ALS, EEG has shown abnormal resting-state brain activity in the alpha (bvFTD and ALS) (Mai et al., 1998, Nishida et al., 2011, Santhosh et al., 2005), beta (bvFTD) (Lindau et al., 2003) and theta (ALS) (Jayaram et al., 2015, Nasseroleslami et al., 2019) bands. In addition, in both bvFTD and ALS, higher EEG-based resting-state functional connectivity has been shown in the delta, theta and gamma bands (Dukic et al., 2019, Nasseroleslami et al., 2019, Yu et al., 2016). In ALS, higher functional connectivity was mainly found in motor regions and to a lesser extent in non-motor regions (Dukic et al., 2019, Nasseroleslami et al., 2019). Whether these changes occur early in the symptomatic disease course of ALS is unknown (Dukic et al., 2019, Jayaram et al., 2015, Lindau et al., 2003, Mai et al., 1998, Nasseroleslami et al., 2019, Nishida et al., 2011, Santhosh et al., 2005, Yu et al., 2016).

As compared to EEG, MEG has a higher spatial resolution and allows for more detailed studies of global and regional brain (dys)function (Baillet, 2017, Hughes et al., 2011, Pizzo et al., 2019). Oscillatory dynamics as measured with MEG have shown comparable frequency changes across degenerative diseases with a common pathology, including FTD (Sami et al., 2018). Task-related MEG studies have shown cortical dysfunction in *C9orf72* carriers (repeat expansion*;* associated with both ALS and FTD (DeJesus-Hernandez et al., 2011)) prior to the development of motor symptoms, resembling findings in symptomatic ALS patients (Proudfoot et al., 2017). MEG-based resting-state functional connectivity changes in ALS patients were prominent in the posterior cingulate cortex, which has an important role in cognition. These findings show the potential of MEG to provide biomarkers for brain dysfunction in ALS, including non-motor regions (Leech and Sharp, 2014, Proudfoot et al., 2018b).

In addition to cortical changes beyond motor regions, MRI and pathological studies have shown involvement of subcortical areas (amygdala, thalamus and hippocampus) in bvFTD, and to a lesser extent in ALS (Anderson et al., 1995, Atta and Schwartz, 2013, Baillet et al., 2001, Bede et al., 2018, Brettschneider et al., 2013, Deymeer et al., 1989, Tesche, 1996, van der Burgh et al., 2020). MEG is able to detect subcortical activity (Hillebrand et al., 2016a, Pizzo et al., 2019) and could contribute to our understanding of neuronal dysfunction in subcortical areas and the medial temporal lobes in early symptomatic ALS.

In this study, we hypothesized that early symptomatic ALS and advanced bvFTD are two ends of a disease spectrum. We aimed to examine to what extent patterns of resting-state brain dysfunction in cortical and subcortical regions in patients with early symptomatic ALS resemble those of bvFTD. Based on the literature we predicted that, compared to healthy controls, patients with early symptomatic ALS and patients with bvFTD would show a pattern of higher functional connectivity in the theta, delta and gamma bands extending from frontal to posterior (sub)cortical brain regions. Findings may contribute to the existence of a symptomatic ALS-bvFTD spectrum.

## 2 Methods

### 2.1 Participants

ALS patients were prospectively recruited between September 2013 and December 2016. Patients were referred to the tertiary referral centres for ALS in The Netherlands (Amsterdam University Medical Centres (UMC) and UMC Utrecht). ALS diagnosis was probable, probable-laboratory supported, or definite ALS, as defined by the revised El Escorial criteria (Brooks et al., 2000). Cases could be sporadic or familial (with or without a known mutation in genes associated with ALS and/or bvFTD). Since we aimed to include ALS patients early in the symptomatic disease course, disease duration had to be <12 months from symptom onset. All ALS patients had to have an upright forced vital capacity (FVC) > 70% of the predicted value, as described previously (Beeldman et al., 2020). ALS patients who fulfilled Rascovsky criteria for bvFTD (medical history obtained from family members) or who had a clinical diagnosis of one of the language variants of FTD were excluded.

In addition, bvFTD patients (without ALS; positive control group) and healthy controls (HCs; negative control group) were recruited, as described before (Beeldman et al., 2020, Engels et al., 2016). BvFTD patients were diagnosed with possible or probable bvFTD at a tertiary referral centre for dementia (Amsterdam UMC) (Rascovsky et al., 2011). They could be sporadic or familial cases and were included irrespective of disease duration or genotype. HCs were recruited via the Dutch ALS Association and participating patients (spouses, family members, friends). HCs had no history of neurological or psychiatric disease.

The local medical ethical committee of the Amsterdam UMC approved the study, which was performed in agreement with the Declaration of Helsinki. Written informed consent was obtained from all participants at inclusion.

### 2.2 MEG data acquisition and pre-processing

MEG recordings were obtained prior to an MRI brain scan. All MEG recordings were performed in a magnetically shielded room (Vacuumschmelze GmbH, Hanau, Germany) using a 306-channel whole-head Vectorview system (Elekta Neuromag Oy, Helsinki, Finland). The recording protocol consisted of 5 min of eyes-closed task-free resting-state condition followed by 2 min eyes-open, and again 5 min eyes-closed. All recordings were performed with the participant in supine position. The recordings were sampled at 1250 Hz, with an online anti-aliasing (410 Hz) and high-pass (0.1 Hz) filter.

Malfunctioning channels were excluded after visual inspection of the neurophysiological signals and after applying the extended Signal Space Separation method (xSSS (van Klink et al., 2017)); implemented in the research module of Elekta MaxFilter version 3.0, not commercially available). The number of excluded channels varied between 1 and 12. Artefacts were subsequently removed with the temporal extension of SSS (tSSS) in MaxFilter software (Elekta Neuromag Oy, version 2.2.15) (Taulu and Hari, 2009, Taulu and Simola, 2006), using a subspace correlation limit of 0.9 and window length of 10 s. The head position relative to the MEG sensors was recorded continuously using signals from five head localization coils. The coil positions were digitized, as well as the outline of the participant’s scalp (∼500 points), using a 3D digitizer (Fastrak, Polhemus, Colchester, VT, USA). This scalp surface was used for co-registration with the participant’s MRI scan using a surface matching approach.

MRI data were obtained using a 3.0 T Ingenia scanner (Philips, Best, The Netherlands) with a 32-channel receive-only head coil. For anatomical reference, a high-resolution 3D T1 scan was obtained (1 mm^3^ resolution). Visual inspection of the co-registration between MEG and MRI scalp surfaces was performed for all participants. The co-registered MRI was spatially normalized to a template MRI (Hillebrand et al., 2012). The Automated Anatomical Labelling (AAL) atlas (Tzourio-Mazoyer et al., 2002) was subsequently used to label the voxels in 78 cortical and 12 subcortical regions of interest (ROIs), and transformed back to native space (Gong et al., 2008, Tzourio-Mazoyer et al., 2002). Each ROI’s centroid was used as representative for that particular ROI (Hillebrand et al., 2016b). Beamforming was used to project broad-band (0.5–48 Hz) filtered sensor signals to these centroid voxels (Hillebrand et al., 2012). For computation of the beamformer weights (Hillebrand and Barnes, 2005, Hillebrand et al., 2005), the sphere that best fitted the scalp surface obtained from the co-registered MRI was used as volume conductor model, together with an equivalent current dipole model (with optimum orientation determined using singular value decomposition (Sekihara et al., 2004)) and the broad-band data covariance matrix, which was based on 255 s of data on average (range 127–325). Projection of the sensor-level MEG data through the normalized beamformer weights (Cheyne et al., 2007) resulted in broadband time-series for each centroid of the 90 ROIs.

For each subject, eight non-overlapping artefact-free epochs, each with a length of 16,384 samples (13.1072 s), were selected out of the first 5 min eyes-closed recording. Eyes-open recordings were not analysed due to artefacts and reduced power of the alpha rhythm.

Epoch selection was based on the following criteria using a strict semi-automatic procedure implemented in Matlab, version 2018b: 1) discard epochs with extreme values in the temporal domain. Epochs where the signal amplitude of one or more channels exceeds the average range could be corrupted by artefacts. Typical artefacts were due to (eye) movements, swallowing or dental prosthetics; 2) discard epochs with individual alpha frequency values outside the range mean ± 2 standard deviation (SD) (mean and SD values computed over all the epochs of a single subject), as these could be indicative of drowsiness (Hari and Puce, 2017); and 3) select the ten epochs out of the remaining epochs with the highest individual alpha frequency and relative alpha1 power in occipital channels for that subject, to avoid possible drowsiness biases across subjects. Two examiners (RG and NS) independently evaluated the ten selected epochs: if the epochs (still) contained artefacts then they were excluded. This procedure allowed to select an approximatively equivalent amount of data for each subject in an objective way and led to at least eight non-overlapping artefact-free epochs for each subject. Epochs were converted to ASCII-format and imported into the BrainWave software package, version 0.9.152.12.26 (C.J. Stam; home.kpn.nl/stam7883/brainwave.html).

### 2.3 MEG data analyses

Peak frequency, relative power and functional connectivity (corrected version of the amplitude envelope correlation (AECc)) were computed for each epoch separately and subsequently averaged over epochs. For the spectral analyses, four short epochs of 4096 samples were taken from each long epoch of 16,384 samples, resulting in 32 short epochs. For each epoch, and each ROI, the power spectrum was computed, and the data were filtered in different frequency bands using the discrete Fast Fourier Transform (FFT). The eight long epochs were then downsampled by a factor 4 and used for functional connectivity (AECc) analyses.

The peak frequency was estimated as the frequency at which the power was at its maximum within the 4 –13 Hz band. Relative and absolute spectral power were calculated for the following frequency bands: delta (0.5–4 Hz), theta (4–8 Hz), alpha1 (8–10 Hz), alpha2 (10–13 Hz), beta (13–30 Hz), and gamma (30–48 Hz); the absolute power in each band was divided by the broad-band (0.5–48 Hz) power in order to obtain the relative power. The alpha band was divided in an upper and lower band as these bands are involved in different cognitive processes (Klimesch et al., 1999). We estimated the global relative power and peak frequency by averaging over all ROIs. Absolute power was used to aid the interpretation of group differences in relative power.

Frequency-band specific functional connectivity was estimated using the AECc, an implementation of the AEC (Bruns et al., 2000), corrected for volume conduction/field spread, using a symmetric orthogonalisation procedure (Brookes et al., 2012, Hipp et al., 2012), and normalised to the range (0–1) with 0.5 indicating no functional connectivity (Briels et al., 2020). The AECc was calculated for all possible pairs of ROIs, leading to a 90 × 90 adjacency matrix. Regional functional connectivity for each ROI was estimated as the average functional connectivity of a ROI with the rest of the brain (i.e., averaging over a column in the matrix), and global functional connectivity was estimated as the average functional connectivity over all ROIs.

### 2.4 Statistical analysis

Demographic data and disease variables were summarized as mean and standard deviation (SD) in case of normal distribution or as median and range. Group differences between ALS, bvFTD and HCs groups were analysed using Kruskal-Wallis test. When statistically significant group differences were found, Mann-Whitney U test was used to analyse differences between two groups. χ^2^ test was used for nominal variables.

Global (cortical and subcortical) peak frequency, relative power and functional connectivity within specific frequency bands were summarized as mean and SD and were compared across groups using the Kruskal-Wallis test, with correction for multiple comparisons using the false discovery rate (FDR < 0.05; six frequency bands) (Benjamini and Hochberg, 1995). When statistically significant group differences were found, Mann-Whitney U test was performed to analyse differences between two groups to find out which group was driving this effect.

If there was a significant global effect in peak frequency, relative power or functional connectivity, regional values were post-hoc compared between groups using a Mann–Whitney U test in order to find out which regions were driving this global effect.

Statistical analyses were performed in SPSS, version 26. A two-sided *p*-value below 0.05 was considered statistically significant.

## 3 Results

### 3.1 Participants

Thirty-four ALS patients, 18 bvFTD patients and 18 HCs were included. Characteristics of the participants are summarized in Table 1.

**Table 1:** **Participant characteristics and disease variables**.

	ALS N = 34	BvFTD N = 18	HCs N = 18	*p*-value
Age (years)	64.0 (8.5)	63.9 (10.8)	60.9 (10.0)	0.486
Male (n, %)	24 (71)	14 (78)	9 (50)	0.178
Education (years)	14 (6–18)	14.5 (10–18)	14 (10–18)	0.268
Disease duration (months)	9.0 (5–15)	38 (18–168)	n/a	**<0.001**
Site of onset (l/b/lb)	18/14/2	n/a	n/a	n/a
ALSFRS-R score	40.4 (4.4)	n/a	n/a	n/a
ALSFRS-R slope	0.73 (0.30–2.43)	n/a	n/a	n/a
FVC (%pred)	94.5 (16.6)	n/a	n/a	n/a
*C9orf72* mutation^†^ (n, %)	3 (9)	3 (17)	n/a	0.203
Riluzole^††^ (n, %)	34 (100)	n/a	n/a	n/a

Data are expressed as mean (SD) or median (range), as appropriate. ALS: amyotrophic lateral sclerosis; bvFTD: behavioural variant frontotemporal dementia; HCs: healthy controls; site of onset (l/b/lb): limb onset/bulbar onset/limb and bulbar onset; ALSFRS-R: ALS functional rating scale revised, a score of 48 indicates no physical impairment; ALSFRS-R slope: calculated as 48 - ALSFRS-R score at time of MEG scan divided by the number of months between first symptoms and MEG scan. FVC %pred: forced vital capacity in upright position, percentage of predicted value; n: designates number; n/a: not applicable. ^†^*C9orf72* mutation status was missing in 6 ALS patients and 7 bvFTD patients. ^††^Median treatment duration was 12 weeks (5–39); two patients were treated for 5 and 7 weeks, respectively; all other patients were treated for at least 8 weeks.

### 3.2 Resting-state brain activity

#### 3.2.1 Global peak frequency

**Fig. 1:**
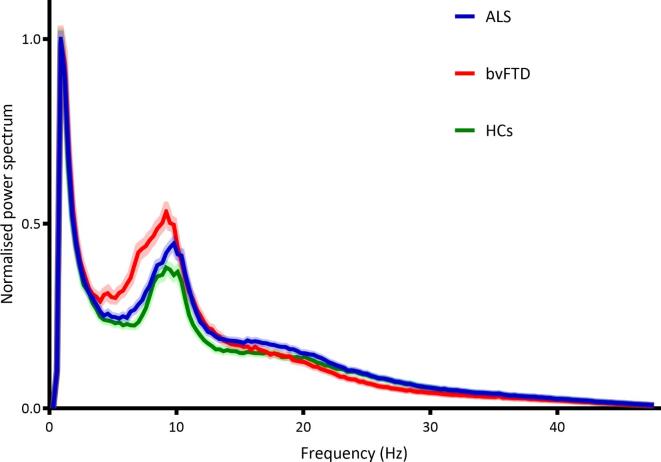
**Power spectra for the different groups.** Normalised power spectra (±standard error of the mean (shaded area)), averaged over 78 cortical and 12 subcortical ROIs for each group between 0.5 and 48 Hz. Abbreviations: ALS: amyotrophic lateral sclerosis; bvFTD: behavioural variant frontotemporal dementia; HCs: healthy controls.

There was no significant difference in global peak frequency between the three groups (H(2) = 0.291, *p* = 0.865; Fig. 1).

#### 3.2.2 Global power differences between groups

**Fig. 2:**
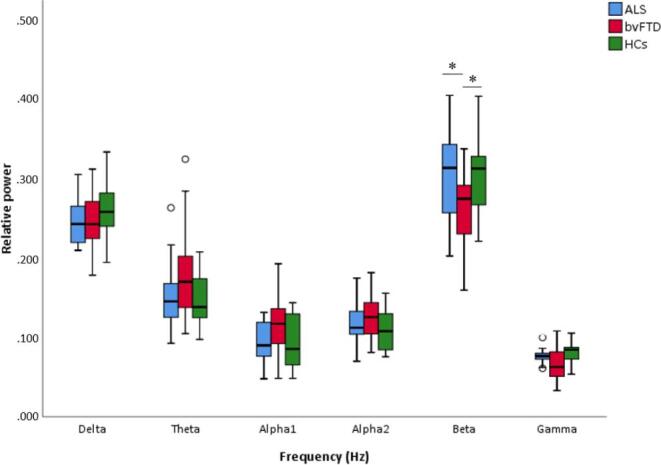
**Global relative power in six frequency bands for the different groups.** Global relative power (i.e., averaged over 78 cortical and 12 subcortical ROIs for each group). * Indicates a significant difference based on Mann-Whitney U scores between bvFTD vs ALS and bvFTD vs HCs. Abbreviations: ALS: amyotrophic lateral sclerosis; bvFTD: behavioural variant frontotemporal dementia; HCs: healthy controls.

Considering individual frequency bands, relative beta band power showed a group effect (Fig. 2; H(2) = 8.513, *p* = 0.008). Post-hoc analyses showed an effect between bvFTD and HCs (U = 88, Z = -2.341, *p* = 0.019) and between bvFTD and ALS (U = 164, Z = −2.731, *p* = 0.006; Fig. 2). An analysis of the absolute beta power confirmed these results by showing lower, albeit not significant, absolute beta power in bvFTD compared to ALS and HCs (bvFTD vs HCs: U = 129, Z = −1.044, *p* = 0.296; bvFTD vs ALS U = 294, Z = -0.231, *p* = 0.817). The group effect for the relative gamma power (H(2) = 7.327, *p* = 0.026) did not survive FDR correction. No significant whole-brain differences between groups were found for the other frequency bands.

#### 3.2.3 Regional beta power differences between groups

**Fig. 3:**
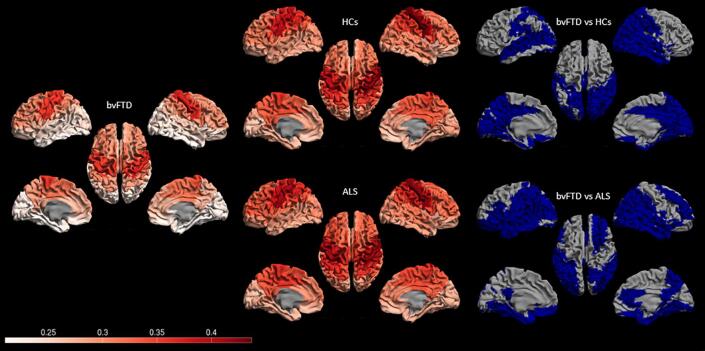
**Regional relative beta power in bvFTD, ALS and HCs and significant differences between bvFTD vs HCs and bvFTD vs ALS.** Regional relative beta power and significant differences (based on Mann-Whitney U scores) between bvFTD vs HCs, and bvFTD vs ALS. Cortical relative beta power distributions for the three groups (group-average) are displayed as color-coded maps on parcellated template meshes on the left side for bvFTD, ALS and HCs; regions corresponding to significant group differences (bvFTD vs HCs and bvFTD vs ALS) are represented in blue on the right cortical meshes which represent regions with a lower beta activity. Note that only cortical regions are displayed (see supplemental Table A for details). Abbreviations: ALS: amyotrophic lateral sclerosis; bvFTD: behavioural variant frontotemporal dementia; HCs: healthy controls.

BvFTD patients showed a lower relative beta power in the temporal lobe (10 regions), parietal lobe (6 regions), occipital lobe (12 regions), limbic lobe (5 regions) compared to HCs (Fig. 3; Supplemental Table A). In addition, group differences between bvFTD patients and HCs were present in nearly all subcortical areas (Supplemental Table A). Compared to ALS patients, bvFTD patients showed a lower relative beta power in the frontal lobe (11 regions), temporal lobe (12 regions), parietal lobe (9 regions), occipital lobe (11 regions), limbic lobe (6 regions) and all 12 subcortical regions (Fig. 3; Supplemental Table A).

### 3.3 Resting-state functional connectivity

#### 3.3.1 Global functional connectivity

Considering individual frequency bands, functional connectivity showed a group effect in the delta and gamma band (Table 2; delta H(2) = 19.216, *p* = <0.001; gamma H(2) = 10.488, *p* = 0.005; Supplemental Figure A).

**Table 2:** **Functional connectivity per frequency band**.

	ALS N = 34	BvFTD N = 18	HCs N = 18
**Delta**	**0.508 (0.008)**	**0.520 (0.016)**	**0.504 (0.004)**
Theta	0.515 (0.010)	0.518 (0.014)	0.509 (0.010)
Alpha1	0.519 (0.015)	0.516 (0.018)	0.517 (0.010)
Alpha2	0.522 (0.012)	0.517 (0.008)	0.515 (0.009)
Beta	0.520 (0.010)	0.515 (0.007)	0.516 (0.008)
**Gamma**	**0.506 (0.009)**	**0.507 (0.012)**	**0.501 (0.002)**

Global functional connectivity (i.e. averaged over 78 cortical and 12 subcortical regions) showed a group effect in delta and gamma band (shown in bold; average and standard deviation; p < 0.05, FDR corrected). Abbreviations: ALS: amyotrophic lateral sclerosis; bvFTD: behavioural variant frontotemporal dementia; HCs: healthy controls.

Post-hoc analyses showed a significant higher delta band functional connectivity between ALS and HCs (U = 198, Z = −2.077, *p* = 0.038), bvFTD and HCs (U = 28, Z = −4.240, *p* < 0.001) and bvFTD and ALS (delta U = 151, Z = −2.981, *p* = 0.003). Post hoc analysis in the gamma band showed a higher functional connectivity ALS compared to HCs (U = 148, Z = −3.039, *p* = 0.002) and in bvFTD compared to HCs (U = 79, Z = −2.626, *p* = 0.009).

#### 3.3.2 Regional functional connectivity

For the two frequency bands that showed significant group effects in global functional connectivity (delta and gamma), we performed post-hoc regional analyses in order to find out which regions were driving this global effect.

#### 3.3.3 Regional delta band functional connectivity

**Fig. 4:**
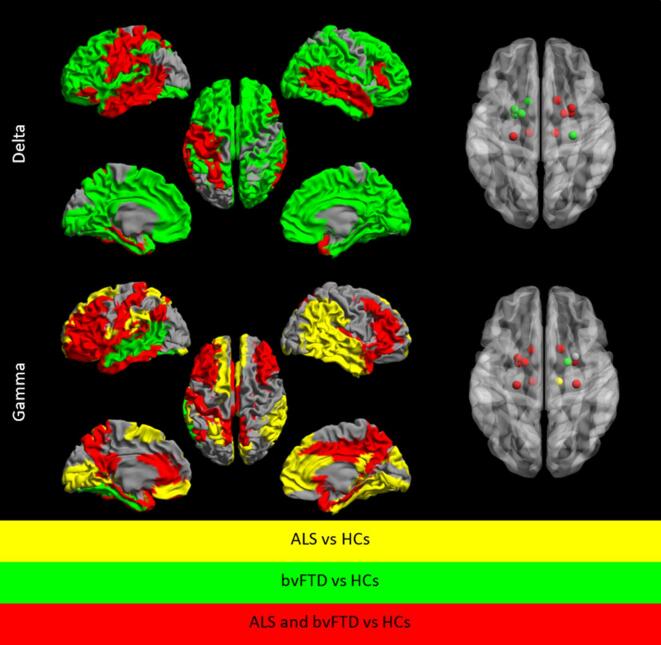
**Significant differences in higher regional resting-state functional connectivity between ALS, bvFTD and HCs in the delta and gamma bands.** Differences in cortical regional resting-state functional connectivity displayed as color-coded maps on a parcellated template mesh, and as color-coded nodes for subcortical regions. Significant higher functional connectivity between groups (based on Mann-Whitney U scores) are displayed as ALS versus HCs (yellow) and bvFTD versus HCs (green). Regions that showed a higher functional connectivity in both ALS and bvFTD patients compared to HCs are shown in red (see supplemental Table B for details). Abbreviations: ALS: amyotrophic lateral sclerosis; bvFTD: behavioural variant frontotemporal dementia; HCs: healthy controls. (For interpretation of the references to color in this figure legend, the reader is referred to the web version of this article.)

ALS and bvFTD showed a higher delta band functional connectivity in the frontal lobe (2 regions), temporal lobe (4 regions), limbic lobe (3 regions) and 7 subcortical regions (left and right thalamus, right globus pallidus, right putamen, right caudate, right amygdala and left hippocampus) compared to HCs. In addition, bvFTD patients showed widespread higher functional connectivity compared to HCs (Fig. 4, Supplemental Table B).

#### 3.3.4 Regional gamma band functional connectivity

In both ALS and bvFTD, gamma band functional connectivity was higher in the frontal lobe (11 regions), temporal lobe (2 regions), parietal lobe (3 regions), limbic lobe (11 regions) and 9 subcortical regions (left and right hippocampus, left and right amygdala, left and right caudate and left putamen, left globus pallidus and left thalamus) compared to HCs. ALS patients also had higher gamma functional connectivity in 6 frontal regions, 5 regions in the temporal lobe, 5 regions in the parietal lobe, 5 regions in the occipital lobe and 2 limbic regions compared to HCs. BvFTD patients also had a higher gamma band functional connectivity in the middle temporal gyrus and fusiform gyrus compared to HCs (Fig. 4, Supplemental Table B).

## 4 Discussion

We examined whether patterns of resting-state brain activity and functional connectivity in cortical and subcortical regions in patients with early symptomatic ALS resemble those of patients with bvFTD, which represent both ends of a presumed disease spectrum. A higher global resting-state functional connectivity was shown in two frequency bands (delta and gamma) in both early symptomatic ALS patients and bvFTD patients, compared to HCs. Post-hoc analyses showed that global changes were driven by regional connectivity changes in a significant number of overlapping frontal and limbic regions, as well as subcortical brain areas.

### 4.1 Resting-state brain activity

Based on findings of reduced alpha and theta power in EEG studies in ALS (Dukic et al., 2019, Jayaram et al., 2015, Mai et al., 1998, Nasseroleslami et al., 2019, Santhosh et al., 2005), we anticipated alterations in global power in these frequency bands using MEG, which were, however, not found. We examined ALS patients early in the symptomatic disease course, reflected by a relatively high ALS-functional rating scale-revised (ALSFRS-R) indicating less motor impairment, and relatively high forced vital capacity. This may explain the absence of changes in low frequency oscillations in our study, since ALS patients in the above-mentioned EEG studies had longer disease durations and more advanced disease stages (Dukic et al., 2019, Jayaram et al., 2015, Mai et al., 1998, Nasseroleslami et al., 2019, Santhosh et al., 2005).

In our study a group effect for the beta band was found, with lower relative beta power in bvFTD patients compared to both ALS patients and HCs, confirmed by the analyses of absolute power. These results are in line with EEG studies in FTD patients (Lindau et al., 2003, Nishida et al., 2011). In task-related MEG studies in bvFTD patients a correlation between behavioural impairment and beta power and frontotemporal beta-coherence has been reported (Hughes et al., 2018, Hughes and Rowe, 2013). Our observed global and regional changes in the beta band in bvFTD patients (median disease duration of 38 months), in combination with EEG changes in occipital, temporal and sensorimotor regions in the beta bands in ALS patients (median disease duration 58 months) reported by others, suggest that resting state brain changes in the ALS-bvFTD spectrum can predominantly be detected in patients with longer disease durations (Dukic et al., 2019).

### 4.2 Resting-state functional connectivity in ALS

A higher resting-state functional connectivity in ALS patients has been shown in multiple well established brain networks using resting-state fMRI (Proudfoot et al., 2018a). In the current study we confirm, using a more direct measure of brain activity than the fMRI blood oxygenation level-dependent (BOLD) signal, that altered functional connectivity is present in ALS, also early in the symptomatic disease course. Higher resting-state functional connectivity in ALS has been attributed to a loss of intracortical inhibitory influence (Clark et al., 2015). This is based on accentuated cortical beta-desynchronization during movement preparation (MEG) (Proudfoot et al., 2017) and diminished post-movement beta-rebound (EEG) (Bizovičar et al., 2014) in ALS patients. These findings have contributed to the concept that an imbalance of local excitatory and inhibitory influences is important in the pathophysiology of ALS, partly related to the glutamate hypothesis (Turner and Kiernan, 2012). The latter is based on the observation of an excess of glutamate (an excitatory neurotransmitter) in cerebrospinal fluid of ALS patients, and decreased transport of glutamate in the motor cortex and somatosensory cortex (Rothstein et al., 1992, Shaw and Ince, 1997). A PET study with the GABA_A_ receptor ligand flumazenil in ALS patients represents in *vivo* evidence that hyperexcitability through interneuronal dysfunction is associated with cognitive performance in ALS (Wicks et al., 2008). A new finding of our study is that altered functional connectivity occurs early in the symptomatic disease course of ALS and in two frequency bands, which suggests that the presumed imbalance of excitatory and inhibitory influences is an early and widespread pathophysiological feature in the brains of ALS patients.

A plausible hypothesis regarding higher functional connectivity in ALS, might be that this is a direct consequence of neuronal damage (due to the pathological process), as we have shown in a previous simulation study in Alzheimer’s disease (de Haan et al., 2012).

Of note, it is unlikely that our findings were influenced by selection bias towards patients with cognitive or behavioural dysfunction, as the ALS patients were consecutively included irrespective of these symptoms, and ALS patients with overt dementia (bvFTD according to Rascovsky criteria and language variants of FTD) were excluded.

### 4.3 Resting-state functional connectivity in bvFTD, in relation to ALS

The overlapping patterns of altered functional connectivity in the delta and gamma bands in ALS and bvFTD, and the hypothesis that loss of intracortical inhibitory influences is associated with higher functional connectivity in ALS, might raise the question on the pathophysiological basis of higher connectivity in bvFTD patients. A number of studies have addressed if – like in ALS – an imbalance of local excitatory and inhibitory influences plays a role in bvFTD (Alberici et al., 2008, Burrell et al., 2011, Cantone et al., 2014, Geevasinga et al., 2015, Schanz et al., 2016). Hughes and colleagues (2018) reported a reduction in task-related beta power changes during a Go-NoGo task, which points at a lack of inhibition (Hughes et al., 2018). Transcranial magnetic stimulation (TMS) studies on hyperexcitability in *C9orf72* patients with either FTD, ALS or ALS-FTD and asymptomatic *C9orf72* carriers, showed abnormalities in ALS patients and were inconclusive for FTD, possibly due to a low sample size (Schanz et al., 2016). However, other TMS studies, in larger cohorts of patients with FTD and in pre-symptomatic *progranulin* mutation carriers have shown dysfunction of intracortical inhibitory circuits (Benussi et al., 2017, Gazzina et al., 2018). In patients with bvFTD, reduced GABA levels in the right inferior frontal gyrus were found, and reduced GABA and glutamate concentrations in this region were both associated with disinhibition, a key symptom of bvFTD (Murley et al., 2020). Interestingly, a relation between GABA levels and beta generation, as well as the back-propagation of information, has been postulated recently (Adams et al., 2021). In addition, it has been proposed that disruption of behavioural control arises from reduced gamma-band (and cross-frequency theta to alpha) connectivity of the inferior frontal gyrus, cortex, together with increased gamma-band connectivity among pre-supplementary motor area and motor regions (Hughes et al., 2018).

Post mortem human brain studies have suggested mechanisms related to hyperexcitability in ALS patients with dementia, through reduced calbindin D28K. Calbindin is an intracellular calcium binding protein associated with GABAergic interneurons and is thought to prevent hyperpolarization through intracellular calcium buffering (Ferrer et al., 1993).

Further evidence for hyperexcitability in FTD is suggested by mouse studies. A FTD mouse model based on the *CHMP2B* mutation – a mutation that causes both ALS and FTD – has linked FTD-related social behaviours to alterations of Ca^2+^-impermeable AMPA receptors (ligand: glutamate) at excitatory synapses of pyramidal neurons in the prefrontal cortex (Gascon et al., 2014). In another mouse model of FTD with parkinsonism linked to chromosome 17 (FTDP-17), increased GABA_A_ receptor-mediated hyperexcitability was shown (García-Cabrero et al., 2013).

In summary, the altered functional connectivity in bvFTD and ALS compared to HCs in our study may be related to hyperexcitability of frontal neurons and interneurons in both disorders, possibly mediated by GABA, glutamate and calcium regulators. This hypothesis could be investigated in future MEG studies when measures of an imbalance between excitatory and inhibitory signals have been fully developed and validated.

### 4.4 Resting-state functional connectivity in ALS and bvFTD – Limbic and subcortical regions

A new finding of our study is the overlap in gamma band resting-state functional connectivity alterations in the limbic regions, including the amygdalae, between ALS and bvFTD. Changes in the amygdalae (i.e. atrophy) have previously been described in both ALS and bvFTD patients (Anderson et al., 1995, Bocchetta et al., 2021, Pinkhardt et al., 2006).

Our findings further suggest abnormal brain connectivity for the hippocampus and other subcortical regions (i.e. thalamus) in patients with ALS and bvFTD. Abnormal subcortical connectivity as detected by MEG has, to the best of our knowledge, only been described in drug-resistant epilepsy and Parkinson’s disease (Hillebrand et al., 2016a, Pizzo et al., 2019, Sorrentino et al., 2021).

Structural involvement, i.e. atrophy of thalamus and hippocampus in bvFTD and ALS has been shown in MRI studies (Anderson et al., 1995, Bede et al., 2018, Deymeer et al., 1989, van der Burgh et al., 2020). Post-mortem studies of ALS patients have shown phosphorylated 43-kDa TAR DNA-binding protein (pTDP-43) inclusions in subcortical areas and the medial temporal lobe, which are thought to reflect an end-stage of non-motor involvement in ALS (Anderson et al., 1995, Brettschneider et al., 2013). This is based on a presumed pattern of “spread” of pTDP-43 pathology across different brain regions (Brettschneider et al., 2013). Involvement of the hippocampus is thought to be present only in those cases who already have involvement of the motor cortex, bulbar and spinal somatomotor neurons, reticular formation and precerebellar nuclei, prefrontal neocortex and basal ganglia (Brettschneider et al., 2013). Although our finding of higher functional connectivity in subcortical areas in early symptomatic ALS should be interpreted with caution, it may suggest that the hippocampus and other subcortical brain regions can be functionally affected earlier in the disease course. The latter is supported by abnormal post mortem white matter integrity of the prefrontal path in ALS patients on diffusion-weighted imaging. These changes were related to myelin and neurofilament degradation, and were also present in patients without prominent pTDP-43 pathology in the adjacent dentate gyrus (Mollink et al., 2019).

As such, our findings may contribute to the understanding of neuronal dysfunction in subcortical areas and the medial temporal lobes and its (temporal) relations to structural imaging and pathological changes in these regions.

### 4.5 Differences in resting-state functional connectivity between ALS and bvFTD

In addition to overlapping patterns in delta and gamma bands between ALS and bvFTD, we also found differences in alterations of functional connectivity between the two disorders.

The most marked difference was found in the delta band which showed widespread altered functional connectivity in bvFTD patients. This is in agreement with a previous EEG study in bvFTD (Yu et al., 2016), whereas EEG studies in ALS have shown altered connectivity in the delta, theta, and gamma bands, predominantly around the motor cortex (Dukic et al., 2019, Iyer et al., 2015, Nasseroleslami et al., 2019). One explanation of the less pronounced delta band connectivity changes in our early symptomatic ALS cohort compared to the bvFTD patients may be that delta band changes occur in later disease stages of neurodegenerative diseases. Support for this observation can be found in patients with Parkinson’s disease in whom quantitative EEG changes in the delta band showed the highest correlation with longitudinal cognitive performance (Caviness et al., 2014).

## 5 Strength and limitations

In addition to its strengths (evaluation of ALS patients early in the symptomatic disease course, inclusion of a disease control group (bvFTD), use of a functional connectivity metric that is corrected for the effects of MEG signal spatial leakage, and use of source-reconstructed MEG, including analysis of subcortical grey matter structures), our study has some limitations.

A limitation of our study is related to the use of Riluzole in all ALS patients. Riluzole is a glutamate inhibitor and has shown to induce a reduction of cortical hyperexcitability in ALS patients. This effect, however, has shown to be transient and was only present for a period of 8 weeks after the start of treatment in a previous study (Geevasinga et al., 2016, Vucic et al., 2013). Only two of our patients were investigated within 8 weeks.

The absence of another positive control group (neurodegenerative disease other than bvFTD or ALS) might be considered a limitation. In order to examine whether overlapping features between ALS and bvFTD are specific to these two disorders, or merely a reflection of a final common pathway of neurodegeneration (Sami et al., 2018), other neurodegenerative disorders should be examined. Lastly, the lack of cognitive and behavioural data, the absence of ALS-bvFTD patients, and the absence of longitudinal data are limitations. Future studies should include network analyses, correlations with cognitive and behavioural data, as well as longitudinal findings.

## 6 Conclusion

We observed that frequency-dependent resting-state functional connectivity changes are present in a considerable number of frontal and limbic, as well as subcortical, brain regions in both early symptomatic ALS and bvFTD patients, as compared to HCs. This study contributes to the understanding of brain dysfunction early in the disease course of ALS patients, and in patients with bvFTD, provides electrophysiological evidence of the disease spectrum of ALS-bvFTD, and may support further development of MEG-based biomarkers for the involvement of non-motor areas in ALS.

### CRediT authorship contribution statement

**Rosanne Govaarts:** Formal analysis, Resources, Validation, Visualization, Writing – original draft, Writing – review & editing. **Emma Beeldman:** Conceptualization, Methodology, Resources, Writing – review & editing. **Matteo Fraschini:** Resources, Writing – review & editing. **Alessandra Griffa:** Formal analysis, Resources, Writing – review & editing. **Marjolein M.A. Engels:** Resources, Writing – review & editing. **Michael A. van Es:** Resources, Writing – review & editing. **Jan H. Veldink:** Resources, Writing – review & editing. **Leonard H. van den Berg:** Resources, Writing – review & editing. **Anneke J. van der Kooi:** Resources, Writing – review & editing. **Yolande A.L. Pijnenburg:** Resources, Writing – review & editing. **Marianne de Visser:** Conceptualization, Methodology, Resources, Supervision, Writing – review & editing. **Cornelis J. Stam:** Conceptualization, Methodology, Writing – review & editing. **Joost Raaphorst:** Conceptualization, Methodology, Resources, Validation, Supervision, Writing – original draft, Writing – review & editing. **Arjan Hillebrand:** Formal analysis, Methodology, Resources, Validation, Supervision, Writing – review & editing.

## Declaration of Competing Interest

The authors declare that they have no known competing financial interests or personal relationships that could have appeared to influence the work reported in this paper.
