# Supplementary material for: Cortical and subcortical changes in resting-state neuronal activity and connectivity in early symptomatic ALS and advanced frontotemporal dementia
Source: Neuroimage Clin. 2022 Feb 12;34:102965. doi: 10.1016/j.nicl.2022.102965 (PMC8867127; doi:10.1016/j.nicl.2022.102965)
Supplement: Supplementary data 1 [file mmc1.docx]

**Supplemental material**

**Table A. Regional beta power**

|  | ALS  (n=34) | bvFTD  (n=18) | HCs  (n=18) | bvFTD vs ALS | bvFTD vs HCs |
| --- | --- | --- | --- | --- | --- |
| Rectus_L | .266 (.048) | .225 (.043) | .255 (.045) | 158 |  |
| Olfactory_L | .275 (.049) | .233 (.044) | .269 (.047) | 155 |  |
| Frontal_Sup_Orb_L | .279 (.046) | .248 (.053) | .267 (.033) | 203 |  |
| Frontal_Med_Orb_L | 278 (.042) | .252 (.043) | .271 (.035) |  |  |
| Frontal_Mid_Orb_L | .288 (.044) | .257 (.051) | .276 (.030) | 199 |  |
| Frontal_Inf_Orb_L | .293 (.044) | .252 (.053) | .279 (.038) | 168 |  |
| Frontal_Sup_L | .317 (.054) | .291 (.058) | .299 (.047) |  |  |
| Frontal_Mid_L | .334 (.068) | .307 (.082) | .315 (.052) |  |  |
| Frontal_Inf_Oper_L | .350 (.070) | .307 (.068) | .335 (.058) | 200 |  |
| Frontal_Inf_Tri_L | .330 (.062) | .290 (.075) | .307 (.043) | 190 |  |
| Frontal_Sup_Medial_L | .304 (.049) | .278 (.052) | .282 (.042) |  |  |
| Supp_Motor_Area_L | .340 (.067) | .318 (.063) | .330 (.072) |  |  |
| Paracentral_Lobule_L | .364 (.086) | .356 (.086) | .359 (.087) |  |  |
| Precentral_L | .386 (.080) | .350 (.069) | .376 (.091) |  |  |
| Rolandic_Oper_L | .334 (.069) | .280 (.066) | .318 (.062) | 169 |  |
| Postcentral_L | .414 (.083) | .359 (.074) | .398 (.095) | 199 |  |
| Parietal_Sup_L | .363 (.076) | .293 (.068) | .359 (.073) | 150 | 85 |
| Parietal_Inf_L | .375 (.073) | .306 (.070) | .371 (.079) | 164 | 89 |
| SupraMarginal_L | .351 (.070) | .295 (.076) | .346 (.077) | 186 |  |
| Angular_L | .336 (.071) | .263 (.081) | .330 (.066) | 145 | 82 |
| Precuneus_L | .334 (.072) | .289 (.058) | .329 (.065) |  |  |
| Occipital_Sup_L | .296 (.076) | .240 (.070) | .307 (.083) | 186 | 85 |
| Occipital_Mid_L | .299 (.088) | .247 (.081) | .293 (.069) | 199 |  |
| Occipital_Inf_L | .284 (.094) | .235 (.068) | .283 (.066) |  | 96 |
| Calcarine_L | .264 (.076) | .228 (.066) | .258 (.074) |  |  |
| Cuneus_L | .276 (.081) | .226 (.069) | .280 (.069) | 196 | 88 |
| Lingual_L | .278 (.076) | .231 (.066) | .274 (.070) | 183 | 94 |
| Fusiform_L | .276 (.081) | .211 (.062) | .279 (.063) | 144 | 67 |
| Heschl_L | .320 (.067) | .256 (.053) | .308 (.069) | 144 | 87 |
| Temporal_Sup_L | .308 (.068) | .247 (.062) | .300 (.068) | 159 | 87 |
| Temporal_Mid_L | .290 (.068) | .237 (.072) | .285 (.053) | 168 | 83 |
| Temporal_Inf_L | .275 (.060) | .223 (.060) | .279 (.056) | 149 | 75 |
| Temporal_Pole_Sup_L | .280 (.045) | .246 (.053) | .274 (.043) | 188 |  |
| Temporal_Pole_Mid_L | .265 (.041) | .226 (.043) | .268 (.045) | 144 | 81 |
| ParaHippocampal_L | .274 (.059) | .217 (.048) | .279 (.058) | 138 | 65 |
| Cingulum_Ant_L | .294 (.054) | .267 (.053) | .283 (.046) |  |  |
| Cingulum_Mid_L | .353 (.084) | .312 (.060) | .338 (.082) |  |  |
| Cingulum_Post_L | .329 (.081) | .280 (.057) | .328 (.066) | 197 | 98 |
| Insula_L | .321 (.057) | .261 (.056) | .303 (.050) | 134 |  |
| Rectus_R | .264 (.047) | .225 (.045) | .256 (.046) | 174 | 99 |
| Olfactory_R | .277 (.052) | .229 (.045) | .266 (.052) | 141 | 98 |
| Frontal_Sup_Orb_R | .277 (.044) | .246 (.046) | .261 (.043) |  |  |
| Frontal_Med_Orb_R | .275 (.041) | .252 (.042)) | .268 (.043) |  |  |
| Frontal_Mid_Orb_R | .280 (.043) | .253 (.050) | .270 (.052) |  |  |
| Frontal_Inf_Orb_R | .283 (.052) | .243 (.053) | .275 (.063) | 177 |  |
| Frontal_Sup_R | .320 (.053) | .289 (.056) | .310 (.059) |  |  |
| Frontal_Mid_R | .329 (.057) | .298 (.066) | .313 (.057) |  |  |
| Frontal_Inf_Oper_R | .346 (.076) | .311 (.078) | .352 (.095) |  |  |
| Frontal_Inf_Tri_R | .315 (.057) | .284 (.068) | .308 (.060) |  |  |
| Frontal_Sup_Medial_R | .320 (.053) | .289 (.056) | .310 (.059) | 198 |  |
| Supp_Motor_Area_R | .343 (.073) | .317 (.063) | .332 (.079) |  |  |
| Paracentral_Lobule_R | .356 (.070) | .339 (.076) | .351 (.105) |  |  |
| Precentral_R | .402 (.087) | .357 (.074) | .402 (.092) |  |  |
| Rolandic_Oper_R | .340 (.073) | .287 (.063) | .334 (.072) | 179 |  |
| Postcentral_R | .426 (.090) | .375 (.074) | .437 (.098) |  |  |
| Parietal_Sup_R | .368 (.084) | .294 (.068) | .358 (.079) | 145 | 95 |
| Parietal_Inf_R | .375 (.087) | .312 (.074) | .371 (.074) | 171 | 96 |
| SupraMarginal_R | .375 (.087) | .310 (.075) | .357 (.064) | 167 |  |
| Angular_R | .342 (.080) | .271 (.081) | .343 (.063) | 151 | 75 |
| Precuneus_R | .332 (.079) | .273 (.068) | .328 (.066) | 175 | 90 |
| Occipital_Sup_R | .298 (.079) | .235 (.076) | .308 (.086) | 174 | 83 |
| Occipital_Mid_R | .296 (.079) | .240 (.078) | .302 (.084) | 178 | 94 |
| Occipital_Inf_R | .286 (.075) | .236 (.061) | .287 (.071) | 201 | 92 |
| Calcarine_R | .272 (.081) | .228 (.072) | .288 (.072) | 203 | 77 |
| Cuneus_R | .272 (.079) | .233 (.072) | .276 (.072) |  | 99 |
| Lingual_R | .274 (.072) | .222 (.067) | .285 (.068) | 179 | 76 |
| Fusiform_R | .276 (.073) | .210 (.059) | .279 (.060) | 137 | 66 |
| Heschl_R | .327 (.071) | .263 (.065) | .321 (.069) | 154 | 87 |
| Temporal_Sup_R | .316 (.072) | .257 (.066) | .303 (.063) | 170 | 97 |
| Temporal_Mid_R | .307 (.076) | .237 (.074) | .308 (.061) | 149 | 72 |
| Temporal_Inf_R | .289 (.059) | .227 (.058) | .283 (.053) | 134 | 70 |
| Temporal_Pole_Sup_R | .280 (.057) | .240 (.050) | .276 (.045) | 195 | 99 |
| Temporal_Pole_Mid_R | .262 (.050) | .227 (.044) | .260 (.053) | 188 |  |
| ParaHippocampal_R | .272 (.056) | .218 (.051) | .278 (.053) | 139 | 64 |
| Cingulum_Ant_R | .297 (.053) | .270 (.050) | .282 (.052) |  |  |
| Cingulum_Mid_R | .342 (.075) | .314 (.064) | .335 (.080) |  |  |
| Cingulum_Post_R | .323 (.083) | .268 (.057) | .329 (.065) | 187 | 81 |
| Insula_R | .314 (.063) | .259 (.054) | .306 (.058) | 157 | 92 |
| Hippocampus_L | .282 (.064) | .223 (.050) | .282 (.061) | 136 | 72 |
| Hippocampus_R | .288 (.065) | .228 (.051) | .281 (.056) | 142 | 74 |
| Amygdala_L | .279 (.054) | .233 (.044) | .272 (.054) | 172 | 96 |
| Amygdala_R | .273 (.058) | .231 (.052) | .266 (.052) | 188 |  |
| Caudate_L | .309 (.057) | .261 (.056) | .300 (.054) | 161 |  |
| Caudate_R | .307 (.059) | .260 (.051) | .300 (.061) | 167 |  |
| Putamen_L | .316 (.059) | .257 (.055) | .296 (.054) | 137 |  |
| Putamen_R | .321 (.065) | .256 (.052) | .306 (.058) | 139 | 92 |
| Pallidum_L | .307 (.060) | .245 (.045) | .297 (.056) | 133 | 77 |
| Pallidum_R | .317 (.064) | .249 (.052) | .300 (.055) | 126 | 86 |
| Thalamus_L | .320 (.065) | .261 (.053) | .319 (.063) | 158 | 79 |
| Thalamus_R | .322 (.070) | .262 (.058) | .317 (.061) | 180 | 81 |

Data are expressed as mean (SD). Differences between groups are shown as Mann-Whitney U scores (last two columns), only scores with a *p* < .05 (uncorrected) are presented. Abbreviations: ALS: amyotrophic lateral sclerosis; bvFTD: behavioural variant frontotemporal dementia; HCs: healthy controls.

**Table B. Regional resting-state functional connectivity**

|  | **Delta** | | | | | | **Gamma** | | | | |
| --- | --- | --- | --- | --- | --- | --- | --- | --- | --- | --- | --- |
|  | ALS  (n=34) | bvFTD  (n=18) | HCs  (n=18) | ALS vs  HCs | bvFTD vs HCs | bvFTD vs  ALS | ALS  (n=34) | bvFTD  (n=18) | HCs  (n=18) | ALS vs  HCs | bvFTD vs HCs |
| Rectus_L | .506 (.009) | .506 (.009) | .501 (.004) |  | 50 | 164 | .506 (.009) | .506 (.009) | .501 (.004) | 203 |  |
| Olfactory_L | .509 (.011) | .509 (.011) | .501 (.003) |  | 27 | 112 | .509 (.011) | .509 (.011) | .501 (.003) | 168 | 53 |
| Frontal_Sup_Orb_L | .506 (.008) | .505 (.009) | .500 (.002) |  | 71 | 183 | .506 (.008) | .505 (.009) | .500 (.002) | 186 | 95 |
| Frontal_Med_Orb_L | .506 (.010) | .503 (.007) | .500 (.003) |  | 56 | 144 | .506 (.010) | .503 (.007) | .500 (.003) | 176 |  |
| Frontal_Mid_Orb_L | .506 (.010) | .507 (.015) | .499 (.003) |  | 47 | 146 | .506 (.010) | .507 (.015) | .499 (.003) | 136 | 81 |
| Frontal_Inf_Orb_L | .508 (.013) | .510 (.015) | .501 (.003) | 191 | 32 | 139 | .508 (.013) | .510 (.015) | .501 (.003) | 189 | 90 |
| Frontal_Sup_L | .505 (.008) | .505 (.008) | .500 (.002) |  | 69 |  | .505 (.008) | .505 (.008) | .500 (.002) | 193 |  |
| Frontal_Mid_L | .508 (.011) | .508 (.013) | .500 (.003) |  | 60 | 163 | .508 (.011) | .508 (.013) | .500 (.003) | 149 | 85 |
| Frontal_Inf_Oper_L | .510 (.015) | .514 (.020) | .501 (.003) |  | 41 | 167 | .510 (.015) | .514 (.020) | .501 (.003) | 173 | 62 |
| Frontal_Inf_Tri_L | .509 (.014) | .512 (.017) | .500 (.004) |  | 45 | 136 | .509 (.014) | .512 (.017) | .500 (.004) | 179 | 70 |
| Frontal_Sup_Medial_L | .503 (.007) | .503 (.007) | .500 (.003) |  | 81 | 190 | .503 (.007) | .503 (.007) | .500 (.003) |  |  |
| Supp_Motor_Area_L | .503 (.005) | .502 (.004) | .500 (.003) |  | 53 | 159 | .503 (.005) | .502 (.004) | .500 (.003) | 192 |  |
| Paracentral_Lobule_L | .503 (.006) | .501 (.004) | .502 (.002) |  |  | 174 | .503 (.006) | .501 (.004) | .502 (.002) |  |  |
| Precentral_L | .507 (.011) | .509 (.016) | .501 (.003) | 163 | 59 |  | .507 (.011) | .509 (.016) | .501 (.003) |  |  |
| Rolandic_Oper_L | .510 (.014) | .512 (.021) | .501 (.004) | 194 | 55 | 186 | .510 (.014) | .512 (.021) | .501 (.004) | 164 |  |
| Postcentral_L | .507 (.011) | .510 (.015) | .501 (.003) | 194 | 66 | 194 | .507 (.011) | .510 (.015) | .501 (.003) | 178 | 83 |
| Parietal_Sup_L | .503 (.005) | .502 (.006) | .500 (.003) | 150 | 42 |  | .503 (.005) | .502 (.006) | .500 (.003) | 192 |  |
| Parietal_Inf_L | .505 (.007) | .508 (.012) | .501 (.003) |  | 95 | 200 | .505 (.007) | .508 (.012) | .501 (.003) |  |  |
| SupraMarginal_L | .508 (.012) | .511 (.016) | .502 (.004) | 120 | 61 |  | .508 (.012) | .511 (.016) | .502 (.004) | 199 |  |
| Angular_L | .504 (.006) | .508 (.011) | .501 (.002) |  |  |  | .504 (.006) | .508 (.011) | .501 (.002) | 184 | 97 |
| Precuneus_L | .504 (.005) | .504 (.009) | .500 (.003) |  | 90 | 191 | .504 (.005) | .504 (.009) | .500 (.003) | 146 | 97 |
| Occipital_Sup_L | .503 (.008) | .509 (.007) | .504 (.007) |  |  |  | .502 (.004) | .501 (.004) | .500 (.003) |  |  |
| Occipital_Mid_L | .503 (.010) | .511 (.012) | .504 (.008) |  |  |  | .503 (.005) | .504 (.007) | .500 (.003) |  |  |
| Occipital_Inf_L | .503 (.005) | .504 (.007) | .500 (.003) |  | 61 | 153 | .503 (.005) | .504 (.007) | .500 (.003) |  |  |
| Calcarine_L | .503 (.005) | .502 (.006) | .500 (.002) |  | 94 |  | .503 (.005) | .502 (.006) | .500 (.002) | 193 |  |
| Cuneus_L | .505 (.009) | .513 (.015) | .504 (.010) |  |  |  | .503 (.005) | .501 (.006) | .501 (.002) |  |  |
| Lingual_L | .504 (.006) | .504 (.008) | .500 (.003) |  | 60 | 195 | .504 (.006) | .504 (.008) | .500 (.003) | 161 |  |
| Fusiform_L | .506 (.009) | .508 (.013) | .501 (.004) |  | 58 | 191 | .506 (.009) | .508 (.013) | .501 (.004) |  | 90 |
| Heschl_L | .509 (.013) | .511 (.016) | .501 (.005) |  | 61 | 170 | .509 (.013) | .511 (.016) | .501 (.005) | 153 |  |
| Temporal_Sup_L | .509 (.013) | .510 (.015) | .501 (.003) |  | 58 | 175 | .509 (.013) | .510 (.015) | .501 (.003) | 170 | 99 |
| Temporal_Mid_L | .507 (.011) | .511 (.015) | .501 (.003) | 170 | 70 |  | .507 (.011) | .511 (.015) | .501 (.003) |  | 85 |
| Temporal_Inf_L | .507 (.011) | .509 (.012) | .501 (.004) | 180 | 66 |  | .507 (.011) | .509 (.012) | .501 (.004) | 193 | 67 |
| Temporal_Pole_Sup_L | .509 (.013) | .510 (.016) | .500 (.003) |  | 52 | 170 | .509 (.013) | .510 (.016) | .500 (.003) | 170 | 76 |
| Temporal_Pole_Mid_L | .509 (.013) | .510 (.015) | .501 (.004) |  | 30 | 160 | .509 (.013) | .510 (.015) | .501 (.004) | 180 | 88 |
| ParaHippocampal_L | .508 (.010) | .510 (.016) | .501 (.004) | 193 | 45 | 174 | .508 (.010) | .510 (.016) | .501 (.004) | 168 | 92 |
| Cingulum_Ant_L | .506 (.011) | .504 (.008) | .500 (.003) |  | 73 | 178 | .506 (.011) | .504 (.008) | .500 (.003) | 173 | 97 |
| Cingulum_Mid_L | .506 (.010) | .506 (.010) | .501 (.003) |  | 58 | 184 | .506 (.010) | .506 (.010) | .501 (.003) |  |  |
| Cingulum_Post_L | .507 (.010) | .508 (.013) | .501 (.004) |  | 31 | 136 | .507 (.010) | .508 (.013) | .501 (.004) | 161 | 93 |
| Insula_L | .511 (.014) | .514 (.020) | .501 (.004) |  | 39 | 176 | .511 (.014) | .514 (.020) | .501 (.004) | 142 | 63 |
| Rectus_R | .506 (.009) | .505 (.009) | .501 (.003) |  | 65 | 197 | .506 (.009) | .505 (.009) | .501 (.003) |  |  |
| Olfactory_R | .506 (.009) | .508 (.012) | .501 (.003) |  | 50 | 140 | .506 (.009) | .508 (.012) | .501 (.003) | 203 | 80 |
| Frontal_Sup_Orb_R | .505 (.009) | .504 (.008) | .501 (.003) |  | 84 |  | .505 (.009) | .504 (.008) | .501 (.003) |  |  |
| Frontal_Med_Orb_R | .505 (.010) | .503 (.006) | .500 (.003) |  | 65 | 183 | .505 (.010) | .503 (.006) | .500 (.003) | 167 |  |
| Frontal_Mid_Orb_R | .506 (.009) | .505 (.010) | .501 (.003) |  | 64 | 173 | .506 (.009) | .505 (.010) | .501 (.003) |  |  |
| Frontal_Inf_Orb_R | .508 (.012) | .509 (.017) | .501 (.004) |  | 64 | 169 | .508 (.012) | .509 (.017) | .501 (.004) | 158 | 88 |
| Frontal_Sup_R | .505 (.010) | .505 (.013) | .500 (.003) |  | 90 |  | .505 (.010) | .505 (.013) | .500 (.003) |  |  |
| Frontal_Mid_R | .506 (.009) | .508 (.015) | .501 (.003) |  | 82 | 174 | .506 (.009) | .508 (.015) | .501 (.003) | 198 | 92 |
| Frontal_Inf_Oper_R | .508 (.013) | .511 (.023) | .502 (.004) |  | 54 | 177 | .508 (.013) | .511 (.023) | .502 (.004) |  |  |
| Frontal_Inf_Tri_R | .508 (.012) | .510 (.021) | .500 (.004) | 172 | 63 | 198 | .508 (.012) | .510 (.021) | .500 (.004) | 129 | 86 |
| Frontal_Sup_Medial_R | .504 (.010) | .504 (.010) | .500 (.003) |  | 65 |  | .504 (.010) | .504 (.010) | .500 (.003) | 198 |  |
| Supp_Motor_Area_R | .504 (.007) | .502 (.003) | .501 (.002) |  | 56 | 153 | .504 (.007) | .502 (.003) | .501 (.002) |  |  |
| Paracentral_Lobule_R | .504 (.009) | .511 (.012) | .503 (.006) |  |  |  | .502 (.006) | .503 (.008) | .501 (.003) |  |  |
| Precentral_R | .504 (.008) | .512 (.014) | .503 (.009) |  |  |  | .505 (.006) | .507 (.017) | .501 (.003) |  |  |
| Rolandic_Oper_R | .509 (.013) | .510 (.021) | .502 (.004) |  | 93 |  | .509 (.013) | .510 (.021) | .502 (.004) |  |  |
| Postcentral_R | .505 (.009) | .505 (.012) | .502 (.003) |  | 98 |  | .505 (.009) | .505 (.012) | .502 (.003) |  |  |
| Parietal_Sup_R | .503 (.008) | .510 (.017) | .502 (.009) |  |  |  | .501 (.004) | .503 (.008) | .501 (.002) |  |  |
| Parietal_Inf_R | .505 (.008) | .504 (.009) | .501 (.003) |  | 97 |  | .505 (.008) | .504 (.009) | .501 (.003) | 160 |  |
| SupraMarginal_R | .507 (.010) | .508 (.017) | .502 (.003) |  | 75 | 191 | .507 (.010) | .508 (.017) | .502 (.003) | 182 |  |
| Angular_R | .504 (.005) | .505 (.013) | .501 (.002) |  | 51 | 158 | .504 (.005) | .505 (.013) | .501 (.002) | 191 |  |
| Precuneus_R | .505 (.007) | .504 (.008) | .500 (.003) |  | 74 |  | .505 (.007) | .504 (.008) | .500 (.003) | 166 | 91 |
| Occipital_Sup_R | .502 (.004) | .503 (.006) | .501 (.003) |  | 41 | 129 | .502 (.004) | .503 (.006) | .501 (.003) |  |  |
| Occipital_Mid_R | .501 (.005) | .503 (.008) | .500 (.003) |  | 56 | 117 | .501 (.005) | .503 (.008) | .500 (.003) |  |  |
| Occipital_Inf_R | .502 (.004) | .503 (.006) | .501 (.003) |  | 76 | 138 | .502 (.004) | .503 (.006) | .501 (.003) |  |  |
| Calcarine_R | .504 (.006) | .505 (.009) | .501 (.002) |  | 73 | 165 | .504 (.006) | .505 (.009) | .501 (.002) |  |  |
| Cuneus_R | .503 (.004) | .503 (.005) | .501 (.004) |  | 81 | 150 | .503 (.004) | .503 (.005) | .501 (.004) | 203 |  |
| Lingual_R | .505 (.007) | .505 (.009) | .501 (.003) |  | 74 | 169 | .505 (.007) | .505 (.009) | .501 (.003) | 188 |  |
| Fusiform_R | .505 (.009) | .509 (.016) | .501 (.003) |  | 62 | 170 | .505 (.009) | .509 (.016) | .501 (.003) | 196 |  |
| Heschl_R | .510 (.012) | .509 (.017) | .502 (.004) |  | 56 | 198 | .510 (.012) | .509 (.017) | .502 (.004) | 165 |  |
| Temporal_Sup_R | .510 (.013) | .508 (.018) | .501 (.004) | 189 | 25 | 173 | .510 (.013) | .508 (.018) | .501 (.004) | 178 |  |
| Temporal_Mid_R | .506 (.008) | .507 (.016) | .501 (.003) | 163 | 59 |  | .506 (.008) | .507 (.016) | .501 (.003) | 182 |  |
| Temporal_Inf_R | .506 (.009) | .506 (.015) | .501 (.003) |  | 54 | 180 | .506 (.009) | .506 (.015) | .501 (.003) | 192 |  |
| Temporal_Pole_Sup_R | .509 (.012) | .510 (.018) | .500 (.004) | 182 | 35 | 187 | .509 (.012) | .510 (.018) | .500 (.004) | 151 | 79 |
| Temporal_Pole_Mid_R | .507 (.011) | .508 (.014) | .501 (.003) | 173 | 15 | 149 | .507 (.011) | .508 (.014) | .501 (.003) | 189 | 89 |
| ParaHippocampal_R | .507 (.010) | .510 (.015) | .502 (.002) |  | 44 | 153 | .507 (.010) | .510 (.015) | .502 (.002) | 204 | 77 |
| Cingulum_Ant_R | .506 (.011) | .505 (.011) | .500 (.003) |  | 61 | 176 | .506 (.011) | .505 (.011) | .500 (.003) | 168 |  |
| Cingulum_Mid_R | .506 (.010) | .505 (.006) | .501 (.003) |  | 44 | 163 | .506 (.010) | .505 (.006) | .501 (.003) | 183 | 96 |
| Cingulum_Post_R | .507 (.010) | .508 (.013) | .501 (.003) |  | 38 | 147 | .507 (.010) | .508 (.013) | .501 (.003) | 161 |  |
| Insula_R | .510 (.014) | .512 (.021) | .502 (.004) |  | 49 | 190 | .510 (.014) | .512 (.021) | .502 (.004) | 194 | 92 |
| Hippocampus_L | .508 (.010) | .510 (.013) | .501 (.004) | 178 | 58 | 192 | .508 (.010) | .510 (.013) | .501 (.004) | 149 | 70 |
| Hippocampus_R | .507 (.010) | .509 (.014) | .502 (.003) |  | 48 | 156 | .507 (.010) | .509 (.014) | .502 (.003) | 178 | 81 |
| Amygdala_L | .508 (.012) | .511 (.015) | .501 (.003) |  | 43 | 173 | .508 (.012) | .511 (.015) | .501 (.003) | 166 | 72 |
| Amygdala_R | .508 (.010) | .509 (.016) | .501 (.003) | 193 | 39 | 183 | .508 (.010) | .509 (.016) | .501 (.003) | 171 | 92 |
| Caudate_L | .507 (.011) | .510 (.014) | .501 (.004) |  | 50 | 150 | .507 (.011) | .510 (.014) | .501 (.004) | 188 | 57 |
| Caudate_R | .507 (.011) | .508 (.013) | .501 (.002) | 181 | 23 | 136 | .507 (.011) | .508 (.013) | .501 (.002) | 187 | 77 |
| Putamen_L | .510 (.014) | .513 (.017) | .501 (.004) |  | 38 | 173 | .510 (.014) | .513 (.017) | .501 (.004) | 174 | 52 |
| Putamen_R | .508 (.012) | .511 (.019) | .502 (.003) | 197 | 50 | 160 | .508 (.012) | .511 (.019) | .502 (.003) |  |  |
| Pallidum_L | .509 (.013) | .512 (.016) | .501 (.004) |  | 30 | 156 | .509 (.013) | .512 (.016) | .501 (.004) | 172 | 68 |
| Pallidum_R | .507 (.011) | .510 (.017) | .502 (.003) | 193 | 40 | 165 | .507 (.011) | .510 (.017) | .502 (.003) |  | 85 |
| Thalamus_L | .508 (.011) | .512 (.016) | .501 (.004) | 200 | 44 | 167 | .508 (.011) | .512 (.016) | .501 (.004) | 138 | 59 |
| Thalamus_R | .508 (.011) | .510 (.016) | .502 (.004) | 203 | 51 | 172 | .508 (.011) | .510 (.016) | .502 (.004) | 176 |  |

Data are expressed as mean (SD). Differences between groups are shown as Mann-Whitney U scores, only scores with a *p* < .05 (uncorrected) are presented. Abbreviations: ALS: amyotrophic lateral sclerosis; bvFTD: behavioural variant frontotemporal dementia; HCs: healthy controls.


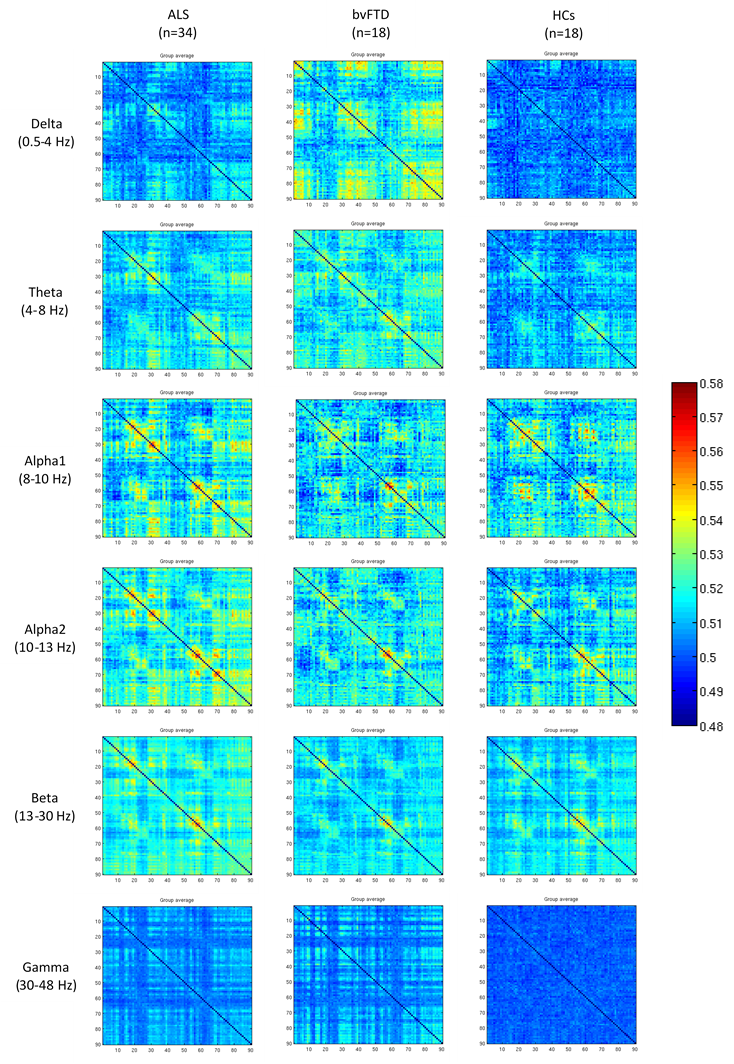


**Figure C. Connectivity matrices of the corrected version of the amplitude envelope correlation (AECc)**

Connectivity matrices averaged across all epochs and subjects. Each row represents a different frequency band (delta, theta, alpha1, alpha2, beta and gamma), and each column shows results for the AECc, for amyotrophic lateral sclerosis (ALS) patients, behavioural variant frontotemporal dementia (bvFTD) patients and healthy controls (HCs). All bands and groups show the matrices with the same colour scale. The ROIs are obtained from the AAL atlas. The matrices are ordered from left to right hemisphere in the following way: rows/columns 1-15 represent left frontal regions, 16-21 left parietal regions, 22-27 left occipital regions, 28-39 left temporal regions, 40-54 right frontal regions, 55-60 right parietal regions, 61-66 right occipital regions, 67-78 right temporal regions, and 79-90 subcortical regions.
